# Medication burden and inappropriate prescription risk among elderly with advanced chronic kidney disease

**DOI:** 10.1186/s12877-020-1485-4

**Published:** 2020-03-04

**Authors:** Clarisse Roux-Marson, Jean Baptiste Baranski, Coraline Fafin, Guillaume Exterman, Cecile Vigneau, Cecile Couchoud, Olivier Moranne, P. S. P. A. Investigators

**Affiliations:** 1 Department of Pharmacy, Nîmes University Hospital, Nîmes, France; 2 Laboratoire Epidemiologie, Santé Publique, Biostatistiques, Université Montpellier, EA2415 Nimes, France; 3 Department of Nephrology, Dialysis and Apheresis, Nîmes University Hospital, Nîmes, France; 4 Nice, France; 5 CHU Rennes, Department of nephrology, 3 rue H le Guilloux, 35000 Rennes, France; 6 INSERM U1085-IRSET, Rennes, France; 7 REIN registry, Agence de la biomédecine, 1 avenue du stade de France, 93212 Saint Denis La Plaine, Saint-Denis, France; 8 Laboratoire Biostatistique Santé Université Claude Bernard Lyon I, UMR CNRS 5558, Lyon, France

**Keywords:** Chronic kidney disease, Elderly, Medication prescription, Polypharmacy

## Abstract

**Background:**

Elderly patients with chronic kidney disease (CKD) frequently present comorbidities that put them at risk of polypharmacy and medication-related problems. This study aims to describe the overall medication profile of patients aged ≥75 years with advanced CKD from a multicenter French study and specifically the renally (RIMs) and potentially inappropriate-for-the-elderly medications (PIMs) that they take.

**Methods:**

This is a cross-sectional analysis of medication profiles of individuals aged ≥75 years with eGFR < 20 ml/min/1.73 m2 followed by a nephrologist, who collected their active prescriptions at the study inclusion visit. Medication profiles were first analyzed according to route of administration, therapeutic classification. Second, patients were classified according to their risk of potential medication-related problems, based on whether the prescription was a RIM or a PIM. RIMs and PIMs have been defined according to renal appropriateness guidelines and to Beer’s criteria in the elderly. RIMs were subclassified by 4 types of category: (a) contraindication; (b) dose modification is recommended based on creatinine clearance (CrCl); (c) dose modification based on CrCl is not recommended but a maximum daily dose is mentioned, (d) no specific recommendations based on CrCl: “use with caution”, “avoid in severe impairment”, “careful monitoring of dose is required” “reduce the dose”.

**Results:**

We collected 5196 individual medication prescriptions for 556 patients, for a median of 9 daily medications [7–11]. Antihypertensive agents, antithrombotics, and antianemics were the classes most frequently prescribed. Moreover, 77.0% of patients had at least 1 medication classified as a RIM. They accounted 31.3% of the drugs prescribed and 9.25% was contraindicated drugs. At least 1 PIM was taken by 57.6 and 45.5% of patients had at least one medication classified as RIM and PIM. The prescriptions most frequently requiring reassessment due to potential adverse effects were for proton pump inhibitors and allopurinol. The PIMs for which deprescription is especially important in this population are rilmenidine, long-term benzodiazepines, and anticholinergic drugs such as hydroxyzine.

**Conclusion:**

We showed potential drug-related problems in elderly patients with advanced CKD. Healthcare providers must reassess each medication prescribed for this population, particularly the specific medications identified here.

**Trial registration:**

NCT02910908.

## Background

The increasing prevalence of chronic kidney disease (CKD) particularly affects older and aging populations [1]. The elderly frequently present multiple comorbidities that lead to polypharmacy and render them vulnerable to medication-related problems, including adverse drug reactions (ADRs) [2]. Elderly patients with CKD require many prescription medications to slow the progression of their renal disease, control specific complications, and manage frequent comorbidities [2]. Because both the aging process and kidney disease modify the pharmacokinetic and pharmacodynamic profiles of drugs, the overall incidence of ADRs is 3 to 10 times higher in older adults with CKD than in those without it [3]. Non-adherence to medication, often reported in patients with CKD as in those with other chronic medical conditions, results in lessened therapeutic response, highly variable dosing, and toxicity [4]. Moreover previous studies have reported that 15–67% of prescriptions for patients with impaired renal function contain errors such as inappropriate doses or intervals, contraindications, or precautions related to renally inappropriate medications (RIMs) [5, 6]. In France, Laroche et al. developed a list adapting the North American Beers’ criteria that are widely used to identify potentially inappropriate-for-the-elderly medications (PIMs) [7]. PIMs are defined as medications that should be generally avoided in people aged ≥75 years because they are either ineffective or at risk of medication-related problems and have a safer alternative [8].

Using this list, Laroche et al. showed that the prevalence of PIMs in older patients admitted to a French acute geriatric unit was 66% [9]. Jones et al. reported a PIM rate of 13% among 100 patients in the USA ≥ 70 years with CKD stages 3–5 [10]. Not surprisingly, some studies suggested that inappropriate medication is related to healthcare costs and utilization of healthcare services such as hospitalization, in- and outpatient visits, and emergency department admissions [11]. Most of these studies are from the United States of America and describe various medication-related issues or inappropriate medications in patients with stage 3–4 CKD or on dialysis [2, 6]. Few studies had previously described prescription patterns and inappropriate medication in non-dialysis elderly patients with advanced CKD [1, 12]. No study focused only on non-dialysis elderly patients with eGFR< 20 ml/min/1.73.

The aim of our study is to describe, first, the overall medication profile of patients ≥75 years with advanced CKD (eGFR < 20 ml/min/1.73 m2) from the multicenter French PSPA study (Parcours de Soin Personnes Agées in French, that is, Care Pathway of the Elderly), and second, which RIMs and PIMs are used most.

## Methods

### Population and data collection

The PSPA multicenter prospective study set up a cohort of patients ≥75 years with advanced CKD.

Patients recruited by nephrologists at 24 nephrology centers throughout France (listed in the appendix as collaborators) met the following inclusion criteria: older than 75 years, CKD with eGFR < 20 ml/min/1.73 m2 (estimated by the sMDRD formula), without a prior kidney transplant, and at least one nephrology clinic visit before enrollment. Consecutive inclusion of in- and outpatients took place over 4 months in each center, with all patients included within 1 year (2009–2010) [13].

Exclusion criteria were acute renal failure and late referral, defined by dialysis starting without previous nephrologist follow-up. The inclusion questionnaire asked for demographic data, clinical conditions (primary renal disease, comorbidities and disabilities), mobility (ability to walk with or without help), living independently in the community or not, and laboratory data. The Institutional Review Board and Ethics Committee of the research institution (Nîmes University Hospital Center), as well as two national bodies, the National Data Protection Authority (CNIL) and the Consultative Committee on the Treatment of Information on Personal Health Data for Research Purposes (CCTIRS), approved the study, which was registered as clinical trial number NCT02910908.

A nephrologist collected active prescriptions at the inclusion visit from the patients. Over-the-counter medications, pro re nata, and self-medication were not collected. The specific information collected for each drug included: drug, brand name, route of administration (transdermal, intravenous, subcutaneous, oral, inhalation, suppository, or eye drops) and dosage. Drugs were coded according to the WHO (World Health Organization) Anatomical Therapeutic Chemical (ATC) classification system [14].

### Method

We performed a cross-sectional analysis of the medication profiles of the patients included in the PSPA study. First, the medication prescriptions were described for the total population according to ATC classification, and the patient’s characteristics according to the tertile of number of daily medications taken. We focused especially on the use of renin angiotensin system inhibitors (RASi), defined as either angiotensin receptor blockers (ARBs) or angiotensin-converting enzyme inhibitors (ACEi) according to specific comorbidities, such as diabetes, heart failure, and nephropathy.

Second, patients were classified according to their risk of potential medication-related problems, based on whether the prescription was a RIM or a PIM. The RIM group comprises medications with any of renal contraindications, dose adjustments, or precautions/warnings according to the patient’s eGFR, as listed in the French Vidal drug dictionary [14] as well as the database on the GPRwebsite, an online pharmacy reference guide, that updates renal dose adjustment guidelines based on international pharmacokinetic studies [15]. According to these guidelines, RIMs are subclassified by 4 types of category: (a) contraindication; (b) dose modification is recommended based on creatinine clearance (CrCl); (c) dose modification based on CrCl is not recommended but a maximum daily dose is mentioned, (d) no specific recommendations based on CrCl: “use with caution”, “avoid in severe impairment”, “careful monitoring of dose is required” “reduce the dose”.

The PSPA cohort included patients with advanced CKD according to their GFR estimated by the MDRD (Modification of Renal Diet Disease) formula. For drug dosing, KDIGO (Kidney Disease: Improving Global Outcomes), the FDA (Food and Drug Administration), and the EMA (European Medicines Agency) all recommend using an estimated GFR value not adjusted for the body surface area (BSA) [16, 17]. As in pharmacology, the Cockroft-Gault (CG) equation is still recommended to adapt drug dosage; we thus defined RIMs according to the CG equation in ml/min. Moreover, the MDRD formula estimate the mGFR with standardized BSA in mL/min/1.73 m2 while CG formula estimate the creatinine clairance in mL/min without BSA standardization. The PIM group includes medications potentially inappropriate for elderly patients according to Laroche criteria adapting the North American Beers’ criteria [18]. Finally, we described the RIM and PIM medications most frequently prescribed and the patient characteristics associated with their prescription.

### Statistical analysis

Patient characteristics and medications are described for the total population. Normally distributed variables are expressed as means (± SD) and non-normally distributed variables as medians and their interquartile ranges.

Patient characteristics associated with at least one PIM prescriptions, and RIM prescriptions, respectively, were evaluated by univariate and multivariate analysis using standard or polytomous logistic regression analysis.

The variables with *p*-values < 0.20 in the univariate analysis were selected for multivariate analysis. All p-values were two-tailed with < 0.05 defined as statistically significant. Analyses were performed with SAS, version 9.3 (SAS Institute, www.sas.com).

## Results

### Study population

Between 2009 and 2010, 573 patients were included in the PSPA study, and baseline medication prescriptions were available for 556 [13]. Mean age at inclusion was 82 years, 57% were men, and median eGFR was 14.2 [11.0–16.7] ml/min/1.73 m2 with MDRD and 13 [10.1–15.4] ml/min with the CG formula. These 556 patients together took 5196 separate medications, mainly orally (89.0%). The median number of drugs per day per patient was 9 [7–11], split into 3 tertile groups: 6 [2–7]; 9 [8–10]; and 12 [11–24]. The characteristics of the overall population according to tertile of daily drugs are presented in the Table 1. The tertile 1, 2 and 3 are respectively composed of 155 patients (28%), 210 patients (38%), and 191 patients (34%). Diabetes, overweight, higher proteinuria, and nephropathy were associated with the prescriptions of more oral drugs to take each day in the univariate analysis; after adjustment in the multivariate analysis, only diabetes was associated with more daily oral medications (Table 1).

**Table 1:** Characteristics of the population, overall and acGEIcording to the number of medications taken daily

	Overall population	Tertile groups of number of drug prescriptions daily		
		Tertile 1	Tertile 2	Tertile 3
Number of patients	556 patients	155 (28%)	210 (38%)	191 (34%)
Number of daily drug prescriptions: n [IQR], (range)		6.0 [5.0; 7.0], (2–7)	9.0 [8.0; 10.0], (8–10)	12.0 [11.0; 13.0], (11–24)
Patients’ characteristics				
Age (year): Mean ± SD	82.5 ± 4.8	82.6 ± 5.0	82.6 ± 4.6	82.2 ± 4.9
Male	318 (57%)	94 (61%)	115 (55%)	109 (57%)
Blood pressure (mmHg)				
SBP Median [IQ]	142.0 [130.0;160.0]	140.0 [130.0; 160.0]	142.0 [130.0; 159.0]	143.5 [130.0; 160.0]
DBP Median [IQ]	73.5 [69.0; 80.0]	73.0 [69.0; 80.0]	75.0 [70.0; 80.0]	72.0 [68.0; 80.0]
Body mass index (BMI): (kg/m2)	26.5 ± 5.0	25.7 ± 4.8	26.3 ± 4.9	27.3 ± 5.1^a^
Diabetes	219 (39%)	53 (34%)	68 (32%)	98 (51%)^b^
Chronic heart failure	194 (35%)	52 (34%)	65 (31%)	77 (40%)
Chronic respiratory disease	62 (11%)	16 (10%)	17 (8%)	29 (15%)
Peripheral vascular disease	138 (25%)	39 (25%)	48 (23%)	51 (27%)
Cerebrovascular disease	75 (13%)	22 (14%)	25 (12%)	28 (15%)
Dysrhythmia	155 (28%)	35 (23%)	61 (29%)	59 (31%)
Active malignancy	55 (10%)	16 (10%)	20 (10%)	19 (10%)
Behavioral disorders	53 (10%)	19 (12%)	19 (9%)	15 (8%)
Residence: independently at home	508 (91%)	141 (91%)	195 (93%)	172 (90%)
Mobility: walks unassisted	499 (90%)	136 (88%)	190 (90%)	173 (91%)
Hemoglobin (g/dl) median [IQ]	11.4 [10.4; 12.4]	11.3 [10.2; 12.4]	11.5 [10.5; 12.3]	11.3 [10.4; 12.4]
eGFR (CG) (ml/min) Median [IQ]	13.0 [10.1; 15.4]	25.0 [16.8; 30.9]	23.9 [19.0; 30.4]	25.2 [18.4; 30.1]
eGFR (MDRD) (ml/min/1.73 m2) Median [IQ]	14.2 [11.1; 16.7]	14.3 [10.9; 16.7]	14.4 [11.3; 17.1]	13.8 [10.9; 16.5]
Proteinuria (g/g): n(%)				
< 0.5	174 (31%)	45 (29%)	78 (37%)	51 (27%)
[0.5–1	105 (19%)	40 (26%)	40 (19%)	25 (13%)
≥ 1	212 (38%)	57 (37%)	70 (33%)	85 (45%)
Miss	65 (12%)	13 (8%)	22 (10%)	30 (16%)^a^
Nephropathy				
Vascular	204 (37%)	49 (32%)	93 (44%)	62 (32%)^a^
Diabetic	135 (24%)	31 (20%)	37 (18%)	67 (35%)
Undetermined	99 (18%)	32 (21%)	37 (18%)	30 (16%)
Glomerulopathy	57 (10%)	17 (11%)	25 (12%)	15 (8%)
Tubulointerstitial	61 (11%)	26 (17%)	18 (9%)	17 (9%)

^a^: Tertile1 vs Tertile3, *p*-value < 0.05 in univariate analysis

^b^: Tertile1 vs Tertile3, *p*-value < 0.05 in multivariate analysis

### Patterns of medication use and frequency

The 3 most common ATC medication classes were those for the cardiovascular system, alimentary tract and metabolism, and the blood (ATC Classes C, A, and B, respectively) (Table 2). In total, 98.0% of patients had at least 1 cardiovascular system medication prescribed, followed by drugs for alimentary tract and metabolism, with 82.6% of patients taking at least 1 medication of this class, and 72.3% of patients taking at least 1 blood medication.

**Table 2:** Most common medication classes according to the WHO Anatomical Therapeutic Chemical (ATC) classification system

ATC		Number of medication prescriptions: n (%)	Number of patients with at least 1 prescription of this type: n (%)
Alimentary tract and metabolism (A)		934 (18.0%)	459 (82.6%)
A11	Vitamins	306 (5.89%)	245 (44.1%)
A02	Drugs for acid-related disorders	224 (4.31%)	218 (39.2%)
A10	Drugs used in diabetes	179 (3.44%)	135 (24.3%)
A06	Drugs for constipation	98 (1.89%)	83 (14.9%)
Others	Alimentary tract and metabolism medications	127 (2.44%)	118 (21.2%)
Blood and blood-forming organs (B)		559 (10.8%)	402 (72.3%)
B01	Antithrombotic agents	395 (7.60%)	340 (61.2%)
B03	Antianemic preparations	164 (3.20%)	162 (29.1%)
Cardiovascular system (C)		2031 (39.1%)	545 (98.0%)
C03	Diuretics	431(8.29%)	413 (74.3%)
C08	Calcium channel blockers	316 (6.08%)	310 (55.7%)
C10	Lipid-modifying agents	286 (5.50%)	276 (49.6%)
C09	Agent acting on the renin-angiotensin system	281 (5.41%)	262 (47.1%)
C07	Beta-blocking agents	268 (5.16%)	264 (47.5%)
C02	Antihypertensive drugs	203 (3.91%)	166 (29.9%)
Others	Antihypertensive medications	246 (4.73%)	196 (35.3%)
Musculoskeletal system (M)			
M04	Antigout preparations	171 (3.29%)	167 (30.03%)
Others	Musculoskeletal system medications	34 (1%)	165 (29.7%)
Nervous system (N)		450 (8.70%)	273 (49.1%)
N05	Psycholeptics	212 (4.08%)	184 (33.1%)
N06	Psychoanaleptics	95 (1.83%)	82 (14.8%)
N02	Analgesics	110 (2.12%)	94 (16.9%)
Other	nervous system medications	33 (0.64%)	32 (5.8%)
Various (V)		397 (7.6%)	296 (53.2%)
V03	Drugs for treatment of hyperkalemia and hyperphosphatemia	122 (2.35%)	122 (21.94%)
		275 (5.29%)	242 (43.53%)
Other class of medications		620 (11.9%)	401 (72.1%)

Antihypertensive treatments were common, with 97.0% of patients prescribed at least 1 medication of this type. Diuretics were widely prescribed, with 74.3% of patients taking at least 1 medication. At least 1 RASi (i.e., ARB or ACEi) was prescribed for 47.1% of patients in this overall population of elderly people with advanced CKD, for 53% of the patients with diabetes, 40% of those with chronic heart failure (CHF), and 40% of those with neither diabetes nor CHF (See additional file 1). Drugs for acid-related disorders such as proton pump inhibitors (PPIs) were prescribed to 39.2% of patients. Medications for subcutaneous administration accounted for 390 prescriptions (7.5% of the entire population) for 313 patients (56.3%), including erythropoietin and insulin (64.0 and 30.0% of subcutaneous medication prescriptions, respectively).

**Fig. 1:**
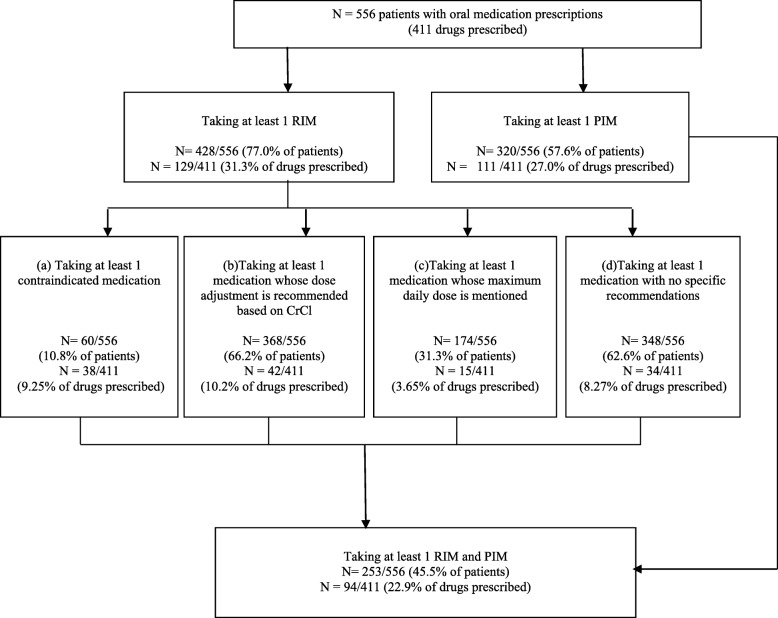
Use of medications classified as renally inappropriate medications (RIMs) and potentially inappropriate-for-the-elderly medications (PIMs)

At least 1 RIM was prescribed to 77.0% of all patients; they accounted 31.3% of the drugs prescribed (Fig. 1):

- At least 1 medication that is contraindicated was prescribed to 10.8% of all patients (9.25% of drugs prescribed) (Fig. 1). Among the contraindicated medications, those most frequently prescribed were rilmenidine (16.5% of patients), rosuvastatin (6.5%), alfuzosin (5.8%), and buflomedil (3.6%) (Table 3).
- At least 1 medication whose the dose modification is recommended based on ClCr was prescribed to 66.2% of all patients (10.2% of drugs prescribed); Allopurinol (30.0% of patients), atenolol (6.1% of patients), acebutolol (5.6% of patients), lorazepam (5.6% of patients) are the drugs the most often prescribed (Table 4).
- At least 1 medication whose the dose modification based on CrCl is not recommended but a maximum daily dose is mentioned was prescribed to 31.3% of all patients (3.65% of drugs prescribed); Ramipril (12.6% of patients), nebivolol (10.1% of patients) and simvastatine (6.7% of patients) are the drugs the most often prescribed (Table 4).
- At least one medication with no specific recommendation of dose modification based on CrCl was prescribed to 62.6% of all patients (8.27% of the drugs prescribed); Bisoprolol (16.7% of patients, clopidogrel (16.4% of patients) and fluindione (12.9% of patients) are the drugs the most often prescribed (Table 4).

**Table 3:** Contraindicated medications prescribed most frequently for the study population, defined by the CG equation

Medications	Patients (%)
Rilmenidine^a^	92 (16.5)
Rosuvastatin	36 (6.5)
Alfuzosin	32 (5.8)
Buflomedil^a^	20 (3.6)
Acarbose	9 (1.6)
Citalopram	9 (1.6)
Fenofibrate	9 (1.6)
Indapamide	9 (1.6)
Spironolactone	9 (1.6)
Colchicine, tiemonium, opium	6 (1.1)

^a^*Rimenidine and buflomedil are both Renally Inappropriate Medications (RIM) and Potentially Inappropriate-for-the-elderly Medication (PIM)*

**Table 4:** Most common mmedications requiring dose adjustment or precautions, defined by the CG equation

Category	Medications	Patients (%)
dose modification is recommended based on ClCr	Allopurinol	167 (30)
	Atenolol	34 (6.1)
	Acebutolol	31 (5.6)
	Lorazepam^a^	31 (5.6)
	Hydroxyzine^a^	27 (4.9)
	Perindopril	20 (3.6)
	Bromazepam^a^	17 (3.1)
	Tianeptine	15 (2.7)
dose modification based on CrCl is not recommanded but a maximum daily dose is mentionned	Ramipril	70 (12.6)
	Nebivolol	56 (10.1)
	Simvastatin	37 (6.7)
no specific recommendations	Bisoprolol	93 (16.7)
	Clopidogrel	91 (16.4)
	Fluindione	72 (12.9)
	Pravastatin	53 (9.5)
	Repaglinide	35 (6.1)
	Candesartan	31 (5.6)
	Warfarin	15 (2.7)
	Clonazepam	14 (2.5)
	Celiprolol	14 (2.5)

*CrCl* Creatinine clearance

^a^*Lorazepam, Hydroxyzine and Bromazepam are both Renally Inappropriate Medications (RIM) and Potentially Inappropriate-for-the-elderly Medication (PIM)*

**Fig. 2:**
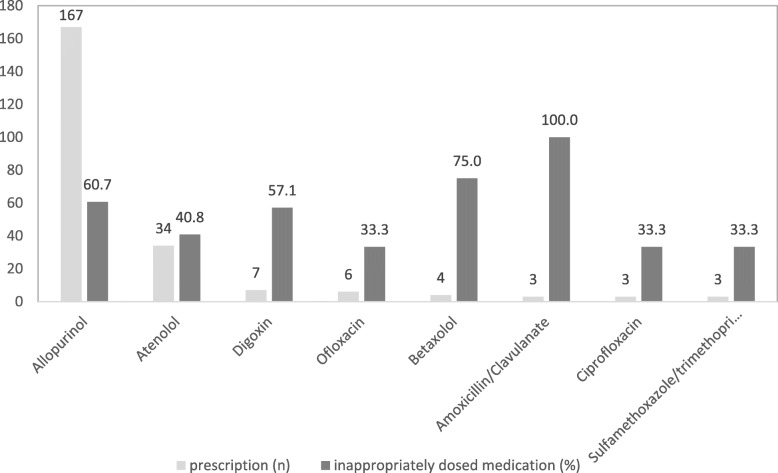
Most frequently medications prescribed at an inappropriate dose for patients with severe chronic kidney disease

Renal misprescribing affected the 39.5% of patients who had at least one medication prescribed at an inappropriate dose. The medications with the most frequently inappropriate doses were allopurinol (60.7% of patients), atenolol (40.8%), digoxin (57.1%), ofloxacin (33.3%), betaxolol (75%), amoxicillin/clavulanate (100%), ciprofloxacin (33.3%) and sulfamethoxazole/trimethoprime (33.3%) (Fig. 2).

At least 1 PIM was prescribed to 57.6% of all patients (Fig. 1). Most PIM were antihypertensive drugs (36% of patients) and sedative or hypnotic drugs (20% of patients). At least 1 medication that is both RIM and PIM was prescribed to 45.5% of all patients. Lorazepam, hydroxyzine, and rilmenidine were the most prescribed PIM that are simultaneously RIM (Table 5). Patient characteristics associated with RIM and PIM in the multivariate analysis are presented in supplementary data (See additional files 2 and 3). RIM is associated with lower BMI, independent living (in the community), lower eGFR, and PIM with higher systolic blood pressure and lower dysrhythmia.

**Table 5:** Common potentially inappropriate medications with renal contraindications, dose adjustments, or precautions for the study population

Medications that are both PIM and RIM	Group of drug	DCI	Patients n (%)
	Central hypertensive drug	Rilmenidine	92 (16.5)
	Central hypertensive drug	Moxonidine	9 (1.6)
	Central hypertensive drug	Clonidine	6 (1.1)
	Central hypertensive drug	Methyldopa	1 (0.2)
	Long-term benzodiazepine	Lorazepam	31 (5,6)
	Long-term benzodiazepine	Bromazepam	17 (3.1)
	Long-term benzodiazepine	Clonazepam	14 (2.5)
	Long-term benzodiazepine	Clorazepate	8 (1.4)
	Long-term benzodiazepine	Lormetazepam	6 (1.1)
	Long-term benzodiazepine	Clobazam	5 (0.9)
	Long-term benzodiazepine	Prazepam	3 (0.5)
	Long-term benzodiazepine	Clotiazepam	2 (0.4)
	Long-term benzodiazepine	Loprazolam	2 (0.4)
	Long-term benzodiazepine	Ethylloflazepate	1 (0.2)
	Anticholinergic drug	Hydroxyzine	27 (4.9)
	Anticholinergic drug	Mirtazapine	9 (1.6)
	Anticholinergic drug	Amitriptyline	4 (0.7)
	Anticholinergic drug	Dexchlorpheniramine	3 (0.5)
	Anticholinergic drug	Alimemazine	2 (0.4)
	Anticholinergic drug	Ropinirole	1 (0.2)
	Vasodilator	Buflomedil	20 (3.6)
	Vasodilator	Pentoxifylline	3 (0.5)
	Other	Digoxin	10 (1.8)
	Other	Opium+Cafeine+paracetamol	5 (0.9)
	Other	Baclofen	1 (0.2)
	Other	Nitrofurantoin	1 (0.2)

## Discussion

We report a complex medical regimen with polypharmacy averagingo9 [7–11] medications prescribed for daily administration in this population ≥ 75 year with advanced CKD and comorbid conditions. Antihypertensive treatment, antithrombotics, and antianemics were the major classes of medication prescribed. Moreover, 77% of these patients with advanced CKD had at least 1 medication prescribed that is either contraindicated or requires dose adjustments/precautions due to advanced CKD; 57.6% of patients had at least 1 PIM and 45.5% has at least one medication that is both RIM and PIM. Renal misprescribing was demonstrated by the 39.5% of patients with at least one medication prescribed at an inappropriate dose. Deprescription is especially important for central antihypertensive drugs, long-term benzodiazepines, and anticholinergic drugs such as hydroxyzine in this population because of the risk of ADR.

We observed more polymedication than Breton et al. [1] who reported an average of 7.1 ± 2.7 medications per day for patients in France with eGFR < 30 ml/min/1.73 m2 and a mean age of 74.3 years ±5.4; they included, however, few patients with eGFR < 30 ml/min/1.73 m2. Other studies have showed that patients with stage 3–5 CKD have 6 to 8 medications per day, while patients with CKD stage 5 treated with dialysis take a median of 12 per day [2, 6]. These studies, however, were conducted in the USA where the national insurance system differs from the French public healthcare system; in the United States of America, insurance companies must approve drug prescriptions, which probably reduces the number taken.

As expected in our population, most medication prescriptions concerned the cardiovascular system and especially antihypertensive treatment due to the high prevalence of hypertension in this population [19]. The prevalence of antihypertensive drugs in our study is 10 to 50% higher than that previously reported among elderly people without advanced CKD and with good blood pressure control [10, 20]. Strict control of blood pressure is recommended for all patients with CKD, according to the latest European Society of Hypertension (ESH) Guidelines [21]. ACEi and ARB medication, that is, RASi, accounted for 47% of the antihypertensive treatment in our study, a level similar to other published results [4, 22]. Other studies have reported a higher level of RASi prescription with the same proportion of diabetic patients, but for younger patients and those with less severe CKD [23, 24]. These ESH therapy guidelines recommend RASi because of their potential to lower blood pressure and reduce albuminuria, as well as their potential benefits in patients with CHF [25]. Yet even in the absence of albuminuria, RASi are recommended to reduce the risk of cardiovascular events [26]. Unsurprisingly, in our population with advanced CKD and diabetes or CHF, the rate of RASi prescription was only around 50%, as these medications are often avoided because of the potential risk of hyperkalemia and acute kidney injury (AKI). With advanced CKD, the benefit of stopping RASi for delaying renal replacement therapy is currently being evaluated in an ongoing interventional study [27].

Patients in our study took numerous medications for metabolic complications, such as drugs to treat hyperphosphatemia and hyperkaliemia, lipid-modifying agents, and vitamins. In later stages of CKD, patients develop numerous metabolic complications that require the prescription of multiple drugs that must comply with guidelines [28]. More surprising is the high rate of pump proton inhibitor (PPI) prescription in our study. Patients with oral anti-thrombotics had a higher frequency of PPI prescription (152/340 (45%) versus 66/216 (30%)). PPIs may be dangerous when used long-term in elderly patients with CKD because of malabsorption of vitamins (B12, iron, calcium, and magnesium, with the associated risks of fractures, enteral infections, and AKI) [29]. Recent studies show that PPI use is associated with a higher risk of CKD, CKD progression, and end stage of renal disease, probably because of repeated, unrecognized AKI and hypomagnesemia [30]. Indications for PPI, especially long-term treatments, must be reassessed for patients with CKD, and preference given to safer alternative, such as antihistaminic H2.

The prevalence of RIM prescriptions in our study (77%) is higher than the prevalence reported in the recent american study by Chang et al. [6], studying patients with CKD stages 3–5. Because of their possible side effects, RIM must usually be reassessed frequently, especially as eGFR worsens; safer alternatives must be chosen if possible, especially for those RIM that are contraindicated. Despite contraindications, some drugs continue to be prescribed, either because of positive benefit/risk ratio or post-marketing studies have demonstrated safety, nor when no alternative exists. For example, rilmenidine could be used at a dose of 1 mg but only if hypertension is uncontrolled with optimum doses of diuretics, ARBs and ACEi, beta blockers, and calcium channel blockers, in combination [31]. However, urapidil, which is a drug that is belonging to the same class of hypertensive agents, is not contraindicated and requires no dose adjustment. Rosuvastatin is the second most contraindicated drug prescribed in our study. The specific hepatic metabolism of this drug means that its plasma concentration is 3 times higher in these patients than in individuals without CKD [32]. An initial 5 mg dose of rosuvastatin with a maximum of 10 mg appears useful, but atorvastatin is a safe alternative [33, 34]. A post-marketing study showed that the use of alfusozin and citalopram appears safe [35, 36]. When no alternatives exist (colchicine for the treatment of acute gout, spironolactone for heart failure), the dose must be as low as possible and reassessed. The other contraindicated drugs used in our study (buflomedil, acarbose, fenofibrate, indapamide) concerned only 9% of our elderly patients and must be reassessed. Allopurinol is the most frequently prescribed medication that requires dose adjustment: it was given at inappropriate doses to two thirds of the patients taking. Medication prescription of allopurinol requires dose adjustment in view of the toxicity of allopurinol in patients with CKD, including rash, gastrointestinal intolerance, leukopenia, and severe hypersensitivity reaction [37]. However, decreasing uric acid levels in hyperuricemic patients with CKD to slow CKD progression is still controversial [37].

It is essential to select the safest drug or to adapt the dosage in order to prevent drug related problem. But little information is available on the aging-related changes and CKD-related changes in pharmakocinetic and pharmacoynamic profile of a number of drugs. In our study, we found that for 8.27% of prescribed drugs (that concerned 62.6% of patients), there is no specific recommendations of dose adjustment. Moreover renal misprescribing affected 39.5% of patients who had at least one medication prescribed at an inappropriate dose. It is important to ensure appropriate doses are prescribed in relation to the level of renal impairment. Therapeutic monitoring is possible for some drugs (for exemple fluindione) but it is not available for all medications. Providers reported poor knowledge of medications requiring dosage adjustment; health information technologies, with computerized alerts could be helpful guide for physician prescribing practice in patient with CKD [38]. But collaborative strategies, such as the routine inclusion of pharmacists in the CKD care model shown more effectiveness [39]. Moreover there is a lack of evidence-based data to guide physicians on precautions and dosage adjustments and a lack of quantitative recommendations in the informations sources [40]. The safety profile of some drugs in case of renal impairment is often based upon knowledge of postmarketing rather than controlled trial. For exemple, for cardiovascular drugs, the information sources often did not provide explicit information for dosage adjustment [41]. Instead of a clear quantitative recommendation, terms like “reduce the dose” or “loading dose should be conservative” were often use. In these cases, we only can recommend some measures to reduce the risk of adverse events: (a) the initiation dose of drugs that require dose adjustment or precautions must be as low as possible, (b) the strategy “start low, go slow” must be preferred, (c) the benefit/risk ratio of each medication must be checked and medication management therapy should regularly be review.

The prevalence of PIM observed in our study is lower than that reported in the USA study by Jones et al. [10], which included 100 patients with CKD stages 3–5 and a mean age of 80 years; instead it is very similar to that reported in other studies focused on elderly patients but without advanced CKD [7, 42]. Pharmacological treatment for elevated blood pressure in advanced CKD patients often requires a combination of several antihypertensive medications, such as central hypertensive drugs, to reach blood pressure (BP) goals [43]. Rilmenidine is often prescribed, but is not recommended to control BP in the elderly or in CKD patients [31]. Other antihypertensive medications should be optimized. Other PIM, such as long-term benzodiazepines, cerebral and peripheral vasodilators, and anticholinergic drugs, are widely prescribed in the elderly without advanced CKD, despite the risks of falls and sedation, particularly in France [7]. Anticholinergic drugs are mainly H1-antihistamines and neuroleptics. H1-Antihistamines are frequently used to treat uremic pruritus but these drugs showed less effectiveness to treat uremic pruritus than gabapentin [44]. Neuroleptics (i.e mirtazapine and alimemazine) are often used for their hypnotic properties instead of more appropriate hypnotic drugs like short- acting benzodiazepines.

Discontinuation of or substitution for drugs that are PIM and RIM in elderly patients with advanced CKD must be considered [45]. Finally, special attention should be paid to patients with higher systolic blood pressure (BP), body mass index (BMI), lower CG-defined GFR, and living independently, as these groups are associated with higher risks of treatment by PIMs or RIMs. There are alternatives to the beer’s criteria or the French list of Laroche such as the STOPP (Screening Tool of Older Person’s potentially Inappropriate Prescriptions) and START (Screening Tool to Alert Doctors to Right Treatment) criteria. These criteria allowed to detect not only misuse but underuse too. Some studies have demonstrated a very low prescription rate of targeted medications within specific CKD patient [23]: for exemple RASi for patients with diabetes and proteinuria, iron and erythropoietin for anaemic patients, statin for patients with coronary artery disease. Our study doesn’t detect this underuse, probably because our patients had at least one consultation with a nephrologist. To our knowledge, there is no specific tool for CKD patients to detect misuse and underuse medications.

Our study has various limitations that should be addressed. First, it may paint an overly rosy picture of overall prescriptions, as all these patients were included in the study by nephrologists, who should already have detected and rectified use of medications that are contraindicated or require dose adjustments. Prescription information was recorded by the nephrologist from the patient’s prescriptions; it is therefore possible that prescriptions by other doctors (for example general practitioners) or self-medication were missed. Use of self medication is current in the Elderly [3]. For example, Nonsteroidal antinflammatory drugs are widely available over the counter and patients use them on their own without advertising their healthcare providers [46]. No medication history was performed during the study. However, medication history should be obtain by the intervention of a clinical pharmacist. It must gather at least 3 sources of information that could be patient’s interview, phone contact with the community pharmacist and/or the general practitioner, review of self-prepared, medication list or personal medical records, review of medication containers, summaries of previous hospitalization or outpatient visits, in order to obtain the mot possible accurate updated list of current medication. In our study daily medication burden were thus probably underestimated. Moreover, because most of the patients included in the study were living independently in the community, these results are difficult to generalize to institutionalized elderly patients. Second, no information is available about medication adherence or about possible ADRs or other events that might have influenced the observed prescriptions. Third, we did not evaluate the risk of ADR associated with medication interactions. The follow-up of the cohort presumably could look at ADRs. Fourth, the generalizability of the results will be restricted to CKD French clinic and need to be validated in non CKD clinic and other health care systems. Finally, we used 2015 recommendations for the qualitative evaluation of medications [47], although our prescriptions come from 2010, and we have no information about practitioners’ changes in prescription practices since then. The follow-up of the cohort presumably could look at ADRs. Otherwise statistical approach used to identify patient’s characteristics associated to PIM and RIM prescription could result in overfitting limiting generalizability of these results. The major strength of this study is the large elderly population with advanced CKD from more than 20 nephrology departments under the care of 70 nephrologists in France, which provides a description of the overall and relatively unknown medication patterns in this particular population.

Our study showed that reassessment of medication prescriptions is needed for elderly patients with advanced CKD. Collaboration and good communication between general practitioners, nephrologists, cardiologists, pharmacists, and geriatricians is required to review the appropriateness of medication prescription [2]. Heath professionals must develop an effective partnership with the patients. Medication review, medication reconciliation, patient counseling, and multidisciplinary meetings are several important approaches to improving medication safety [48] by conducting a benefit-risk analysis for indications, dosage, interactions, side effects, adherence to guidelines for elderly patients and those with advanced CKD. At the end of this medication review, physicians should limit the number of medications as far as possible, but the development of an algorithm ensuring that the highest priority drugs are prescribed in this population is essential. Limiting the number of medications should also improve patient adherence but deprescribing is a real challenge [49, 50]. After this showing of the exposure of elderly people with advanced CKD to both RIM and PIM, further studies must examine the impact of deprescription of these targeted medications.

## Conclusion

In this study, we reported polypharmacy and exposure to potential drug-related problems in elderly patients with advanced CKD. In this population, healthcare providers must be more aware of the need to evaluate the benefit-risk ratio of each medication prescribed, such as the specific medications identified in this study. Collaborative patient-centered approaches with all patients’ health care professionals must be developed. Finally, the development of new medication needs to consider this complex polypharmacy in this growing population.

## Supplementary information

**Additional file 1.** Characteristics of patients with prescriptions for ARBs* or ACE inhibitors** (* ARB: Angiotensin receptor blocker, **ACEi: Angiotensin-converting enzyme inhibitor)

**Additional file 2.** Characteristics of patients according to the Renally Inappropriate Medication (RIM) according to Cockroft Gault formula prescription

**Additional file 3.** Characteristics of patients according to Potentially Inappropriate Medications (PIM) prescription

## Data Availability

The datasets used and/or analyzed during the current study are available from the corresponding author.

## Abbreviations

ACEi: Angiotensin Converting Enzym Inhibitor
ADR: Adverse Drug Reaction
AKI: Acute Kidney Injury
ARB: Angiotensin Receptor Blocker
ATC: Anatomical Therapeutic Chemical classification
BMI: Body Mass Index
BP: Blood Pressure
BSA: Body Surface Area
CCTIRS: Comité Consultatif sur le Traitement de l’Information en Recherche en Santé (French: Consultative Committee on the Treatment of Information on Personal Health Data for Research Purposes)
CG: Cockcroft and Gault
CHF: Chronic Heat Failure
CKD: Chronic Kidney Disease
CNIL: Commission Nationale de l’Informatique et des Libertés (French: National Data Protection Authority)
eGFR: estimated Glomerular Filtration Rate
EMA: European Medicine Agency
ESH: European Society of Hypertension
FDA: Food And Drug Agency
GFR: Glomerular Filtration Rate
KDIGO: Kidney Disease Improving Global Outcomes
MDRD: Modification Of Diet in Renal Disease
PIM: Potentially Inapropriate Medication
PPI: Pump Proton Inhibitor
PSPA: Parcours de Soin Personnes Agées (French: Care Pathway of the Elderly)
RASi: Renin Angiotensin System inhibitors
RIM: Renally Inapropriate Medication
SD: Standard Deviation
SRAi: System Renin Angiotensin Inhibitors
WHO: World Health Organization
