# Supplementary material for: Medication burden and inappropriate prescription risk among elderly with advanced chronic kidney disease
Source: BMC Geriatr. 2020 Mar 4;20:87. doi: 10.1186/s12877-020-1485-4 (PMC7057617; doi:10.1186/s12877-020-1485-4)
Supplement: Supplementary file 1 — Additional file 1. Characteristics of patients with prescriptions for ARBs* or ACE inhibitors** (* ARB: Angiotensin receptor blocker, **ACEi: Angiotensin-converting enzyme inhibitor) [file 12877_2020_1485_MOESM1_ESM.pdf]

Additional file 1. Patients with prescriptions for ARBs or ACE inhibitors***

	Study population	ARB* or ACEi**<br>Number of patients with at least 1 such prescription
**Number (percentage) of patients with CKD and:**	**556 (100%)**	**261 (47%)**
Diabetes: n (%)	***215 (38%)***	***115 (53%)***
Diabetes and heart failure stage I-II: n (%)	53 (9.5%)	29 (55%)
Diabetes and heart failure stage III-IV	28 (5.0%)	13 (46%)
Diabetes and no heart failure	134 (24.1%)	73 (54%)
Chronic Heart Failure: n (%)	***104 (19%)***	***42 (40%)***
Heart failure stage I-II and no diabetes;	65 (11.7%)	26 (40%)
Heart failure stage III-IV and no diabetes	39 (7.0%)	16 (41%)
CKD without heart failure or diabetes: n (%)	***225 (40.5%)***	***104 (40%)***
According to nephropathy		
Vascular	103 (45.2%)	56 (54%)
Cystic	43 (18.9%)	14 (32%)
Chronic glomerulonephritis	30 (13.2%)	11 (37%)
Unknown	49 (21.5%)	23 (47%)

* ARB: Angiotensin receptor blocker

* ACEi: Angiotensin-converting enzyme inhibitor
