# Supplementary material for: Medication burden and inappropriate prescription risk among elderly with advanced chronic kidney disease
Source: BMC Geriatr. 2020 Mar 4;20:87. doi: 10.1186/s12877-020-1485-4 (PMC7057617; doi:10.1186/s12877-020-1485-4)
Supplement: Supplementary file 2 — Additional file 2. Characteristics of patients according to the Renally Inappropriate Medication (RIM) according to Cockroft Gault formula prescription [file 12877_2020_1485_MOESM2_ESM.pdf]

Additional file 2. Characteristics of patients according to prescriptions of renally inappropriate medications (defined by Cockroft Gault formula

**Patient characteristics :**<br>**N = 556 patients**<br>**Overall and according to RIM prescription**<br>**(renal contraindication or dose adjustments)**	All patients<br>N = 556	Patients without RIM medications<br>N = 128 (23%)	Patients with at least one RIM<br>N = 428 (77%)	P<br>Bivariate analysis
Age (year): Mean ±SD	*82.5 ±4.8*	82.6 ± *4.6*	82.5 ± *4.9*	0.79
Male	*318 (57%)*	77 (60%)	241 (56%)	0.44
Blood pressure (mmHg) SBP Median [IQ]<br>DBP Median [IQ]	*142 [130; 160]*<br>*73.5 [69.0; 80.0]*	140 [130; 159]<br>76.0 [70.0; 80.0]	142 [130; 160]<br>72.0[68.0; 80.0]	0.56<br>0.68
Body mass index (BMI):(kg/m2)	*26.5 ± 5.0*	27.4 ± *5.2*	26.2 ± *4.8*	0.01*
Diabetes	*219 (39%)*	49 (38%)	170 (40%)	0.77
Chronic heart failure	*194 (35%)*	43 (34%)	151 (35%)	0.73
Chronic respiratory disease	*62 (11%)*	18 (14%)	44 (10%)	0.23
Peripheral vascular disease	*138 (25%)*	30 (23%)	108 (25%)	0.74
Cerebrovascular disease	*75 (13%)*	15 (12%)	60 (14%)	0.53
Dysrhythmia	*155 (28%)*	35 (27%)	120 (28%)	0.88
Active malignancy	*55 (10%)*	15 (12%)	40 (9%)	0.43
Behavioral disorders	*53 (10%)*	15 (12%)	38 (9%)	0.34
Residence: independently at home	*508 (91%)*	110 (86%)	398 (93%)	0.01*
Mobility: Walks unassisted	*499 (90%)*	110 (86%)	389 (91%)	0.09
Hemoglobin (g/dl) Median [IQ]	*11.4[10.4; 12.4]*	11.4 [10.3; 12.5]	11.4 [10.4; 12.3]	0.62
eGFR (ml/min) Median [IQ]	*13.0 [10.1; 15.4]*	25.6 [20.8; 32.3]	24.3 [17.5; 29.6]	0.003*
Proteinuria (g/g) : n(%)<br><0.5<br>[0.5-1<br>≥1<br>Miss	 *174 (31%)*<br>*105 (19%)*<br>*212 (38%)*<br>*65 (12%)*	 38 (30%)<br>25 (20%)<br>55 (43%)<br>10 (8%)	 136 (32%)<br>80 (19%)<br>157 (37%)<br>55 (13%)	 0.70<br>0.35<br>0.68<br>0.19
Nephropathy Vascular<br>Diabetic<br>Undetermined<br>Glomerulopathy<br>Tubulointerstitial	*204 (37%)*<br>*135 (24%)*<br>*99 (18%)*<br>*57 (10%)*<br>*61 (11%)*	43 (34%)<br>29 (23%)<br>18 (14%)<br>19 (15%)<br>19 (15%)	161 (38%)<br>106 (25%)<br>81 (19%)<br>38 (9%)<br>42 (10%)	0.23<br>0.93<br>0.56<br>0.06<br>0.11

*p<0.05 with multivariate analysis
