# Supplementary material for: Medication burden and inappropriate prescription risk among elderly with advanced chronic kidney disease
Source: BMC Geriatr. 2020 Mar 4;20:87. doi: 10.1186/s12877-020-1485-4 (PMC7057617; doi:10.1186/s12877-020-1485-4)
Supplement: Supplementary file 3 — Additional file 3. Characteristics of patients according to Potentially Inappropriate Medications (PIM) prescription [file 12877_2020_1485_MOESM3_ESM.pdf]

Additional file 3. Characteristics of patients according to prescriptions of potentially inappropriate-for-the-elderly medications (PIM)

**Patient characteristics : N = 556 patients**	All patients N = 556	Patients without PIM Medication N = 236 (43%)	Patients with at least one PIM Medication N = 320(57%)	P Bivariate analysis
Age (year): Mean ±SD	*82.5 ±4.8*	82.7 ± 4.8	82.4 ± 4.83	0.50
Male	*318 (57%)*	140 (59%)	178 (56%)	0.38
Blood pressure (mmHg) SBP Median [IQ]	*142 [130;160]*	140 [125;160]	145 [131;160]	0.009*
DBP Median [IQ]	*73.5 [69.0 ; 80.0]*	72 [70;80]	75 [68;80]	0.43
Body mass index (BMI): (kg/m2)	*26.5 ± 5.0*	26.0 ± 4.3	26.8 ± 5.4	0.08
Diabetes	*219 (39%)*	85 (36%)	134 (42%)	0.16
Chronic heart failure	*194 (35%)*	85 (36%)	109 (34%)	0.63
Chronic respiratory disease	*62 (11%)*	32 (14%)	30 (9%)	0.12
Peripheral vascular disease	*138 (25%)*	56 (24%)	82 (26%)	0.86
Cerebrovascular disease	*75 (13%)*	30 (13%)	45 (14%)	0.58
Dysrhythmia	*155 (28%)*	79 (33%)	76 (24%)	0.01*
Active malignancy	*55 (10%)*	25 (11%)	30 (9%)	0.63
Behavioral disorders	*53 (10%)*	20 (8%)	33 (10%)	0.47
Residence: independently at home	*508 (91%)*	220 (93%)	288 (90%)	0.18
Mobility: Walks unassisted	*499 (90%)*	211 (89%)	288 (90%)	0.73
Hemoglobin (g/dl) median [IQ]	*11.4 [10.4;12.4]*	11.5 [10.5;12.6]	11.3 [10.3;12.2]	0.10
eGFR (ml/min/1.73m2) Median [IQ]	*14.2 [11.1; 16.7]*	14.2 [10.8;16.7]	14.1 [11.3;16.7]	0.92
Proteinuria (g/g): n (%) <0.5	*174 (31%)*	71 (30%)	103 (32%)	0.0049
[0.5-1	*105 (19%)*	57 (24%)	48 (15%)	
≥1	*212 (38%)*	91 (39%)	121 (38%)	
Miss	*65 (12%)*	17 (7%)	48 (15%)	
Nephropathy Vascular	*204 (37%)*	88 (37%)	16 (36%)	0.23
Diabetic	*135 (24%)*	56 (24%)	79 (25%)	
Undetermined	*99 (18%)*	44 (19%)	55 (17%)	
Glomerulopathy	*57 (10%)*	17 (7%)	40 (13%)	
Tubulointerstitial	*61 (11%)*	31 (31%)	30 (9%)	

**p<0.05* with multivariate analysis
